# Comparative Analysis of Maize Physico-Chemical Parameters and Mycotoxin Levels in Dual Environments

**DOI:** 10.3390/toxins16060275

**Published:** 2024-06-17

**Authors:** Bruna Carbas, Sílvia Barros, Andreia Freitas, Ana Sanches Silva, Carla Brites

**Affiliations:** 1 National Institute for Agricultural and Veterinary Research (INIAV), I.P., Av. Da República, Quinta do Marquês, 2780-157 Oeiras, Portugal; bruna.carbas@ipb.pt (B.C.); silvia.barros@iniav.pt (S.B.); andreia.freitas@iniav.pt (A.F.); asanchessilva@ff.uc.pt (A.S.S.); 2 Centro de Investigação de Montanha (CIMO), Instituto Politécnico de Bragança, Campus de Santa Apolónia, 5300-253 Bragança, Portugal; 3 Faculty of Pharmacy, Coimbra, Azinhaga de Santa Comba, University of Coimbra, 3000-548 Coimbra, Portugal; 4 Centre for Animal Science Studies (CECA), University of Porto, 4050-453 Porto, Portugal; 5 GREEN-IT Bioresources for Sustainability, ITQB NOVA, Av. da República, 2780-157 Oeiras, Portugal

**Keywords:** *Zea mays* L., nutritional parameters, fumonisins, deoxynivalenol

## Abstract

Maize (*Zea mays* L.) stands as a vital staple food globally, holding significant nutritional and economic value. However, its susceptibility to mycotoxin contamination under stressful environmental conditions poses a considerable concern. This study aimed to assess the quality and pasting characteristics of maize varieties across two distinct regions and examine the occurrence of mycotoxins influenced by climatic factors. Five maize varieties were cultivated in triplicate in the Golegã and Coruche regions. The nutritional composition (protein, fat, fiber, ash, starch, and lutein), pasting properties, and mycotoxin levels were evaluated. A statistical analysis revealed notable differences in the nutritional profiles of the maize varieties between the two regions, particularly in the protein and lutein content. The peak viscosity ranged from 6430 to 8599 cP and from 4548 to 8178 cP in the maize varieties from the Coruche and Golegã regions, respectively. Additionally, a significant correlation was observed between the climatic conditions and the grain nutritional quality components (*p* < 0.05). The M variety showed the highest ash content, protein content, final viscosity, and setback viscosity and the lowest peak viscosity. The Y variety revealed the lowest fat, fiber, and lutein content and the maximum peak viscosity. The incidence of mycotoxins was notably higher in the varieties from Coruche, which was potentially attributable to higher temperatures and lower precipitation levels leading to more frequent drought conditions. Fumonisin B1 was detected in 58% of the varieties from Coruche and 33% of the samples from Golegã, while deoxynivalenol was found in 87% and 80% of the varieties from Coruche and Golegã, respectively. The H variety, which was harvested in Coruche, exhibited the highest number of fumonisins and higher amounts of protein, lutein, and fat, while fumonisins were not detected in the Golegã region, which was potentially influenced by the precipitation levels. The K variety revealed higher protein and lutein contents, a lower amount of fat, excellent pasting properties (a higher peak viscosity and holding strength and a lower peak time), and no fumonisins B1 or B2. This variety may be considered well adapted to higher temperatures and drier conditions, as verified in the Coruche region. In conclusion, our study underscored the profound impact of environmental factors on the quality and occurrence of mycotoxins in maize varieties.

## 1. Introduction

Maize is one of the most important crops worldwide, with a global production of 1.2 M ton annually. This is around 50% higher than rice and wheat dry grains [1].

Maize is considered a versatile multi-purpose crop; it is used as a livestock feed crop with a varied role as an industrial and energy crop in countries with developed economies, while in countries experiencing economic development, maize is used mainly for human consumption in processed or unprocessed food products [2]. Globally, the use of dry maize grains for feed represents 56% of the production; 13% is used for food; and 20% is used for other non-food uses. However, the consumption of maize as food has increased globally, at around 19 kg/capita/year. It comprises a main element of the human diet and is estimated to account for 42% of the world’s food calories and 37% of the world’s protein intake [1]. 

Mycotoxin contamination, which may occur in the field and/or during storage, is one of the major concerns for maize crops [3]. *Fusarium* spp. and *Aspergillus* spp. are the fungal genera with the most significant impact on maize. The principal mycotoxins found in maize are aflatoxins, fumonisins, zearalenone, ochratoxin A, deoxynivalenol, and trichothecenes [4]. *Fusarium verticillioides* and *F. proliferatum* are the main pathogenic fumonisin-producing species, the *F. graminearum* species produces trichothecenes and zearalenone, and *Aspergillus flavus* is the main aflatoxin-producing species [5]. These species can be harmful to the health of humans, animals, and plants by growing invasively, which is common and lethal in organisms with compromised immune systems, or by contaminating food or feed with mycotoxins [6]. Mycotoxins result in significant financial losses due to mortality, reduced animal productivity, and higher veterinary and human health care expenses (including those associated with cancer, mutagenesis, and estrogenic disorders) [7,8]. When produce with significant contamination is deemed unfit for consumption, it is rejected by the market and ultimately destroyed, resulting in total losses [8]. *Aspergillus* and *Fusarium* species can infect maize prior to harvest and their numbers can increase with poor storage conditions; however, mycotoxin contamination is mainly influenced by environmental conditions [4,9].

Among environmental factors, temperature in particular is the most important factor that affects fungal species prevalence, thus influencing mycotoxin occurrence [10] and maize grain quality [11]. Worldwide, temperatures are expected to rise by 2–5 °C and droughts will become more frequent. These climatic parameters may change in diverse geographical regions [12], and in turn, they could have a profound effect on mycotoxigenic fungi and the production of mycotoxins [13]. Aflatoxin contamination was found in 69% of maize samples, with concentration levels of 1.01–86.1 μg/kg, due to extended periods of hot and dry weather [14]. It has been demonstrated that the *F. verticillioides* infection levels in ripening maize increase in situ when subjected to elevated temperatures during the silking process, with no discernible effects on fumonisin production [15]. However, drought stress increases the occurrence of fumonisins in maize [16]. Climatic change scenarios, higher temperatures (2–5 °C) throughout the world, increases in the total precipitation received by some regions (tropical regions and those at high latitudes), and decreases in others (southern Europe and Africa, and central North America) may result in more favorable weather conditions for mycotoxin occurrence [17].

The nutritional quality of grains is also influenced by the environmental temperature [18]. Heat stress has been found to constrain synthetase activity, leading to a subsequent reduction in the starch content while simultaneously resulting in an increased grain protein content [19]. Drought conditions have also been observed to decrease the starch content while enhancing the crude protein levels, with negligible effects on the fat content. 

Given the significant influence of the climatic conditions (temperature and precipitation) on maize quality and safety, this study aimed to analyze the nutritional quality (ash, fat, protein, fiber, starch, and lutein content) and pasting properties of five maize varieties harvested in two distinct regions within the Tagus Valley region of Portugal. Additionally, the occurrence of mycotoxins, which was influenced by the climatic conditions present in both regions, was assessed.

## 2. Results and Discussion

### 2.1. Nutritional Composition

**Figure 1:**
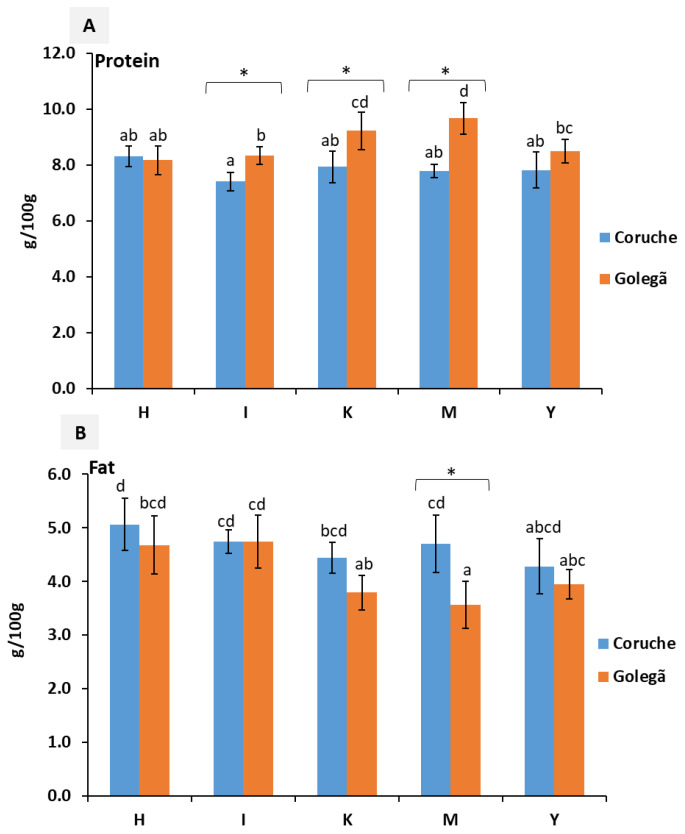
Nutritional composition: (**A**) the protein content and (**B**) the fat content of five maize varieties collected from the Golegã and Coruche regions. The absence of common letters reveals significant differences at *p* < 0.05, and Tukey’s multiple range tests were performed for each year separately. * indicates a significant difference at *p* < 0.05 between the two harvesting regions.

**Figure 2:**
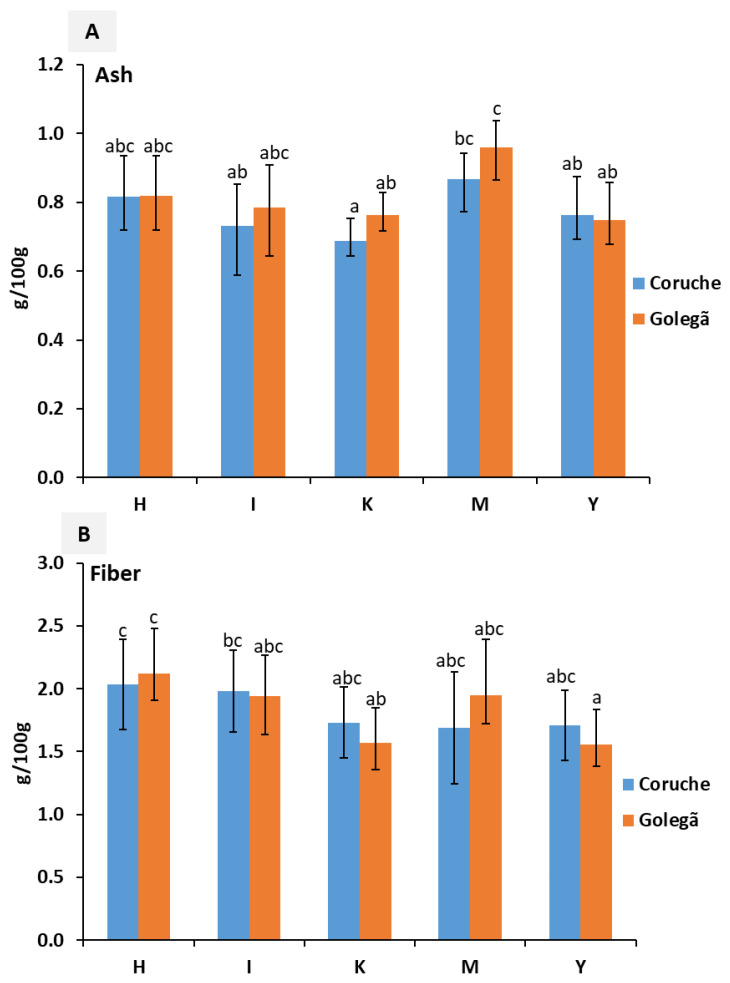
Nutritional composition: (**A**) the ash content and (**B**) the fiber content of five maize varieties collected from the Golegã and Coruche regions. The absence of common letters reveals significant differences at *p* < 0.05, and Tukey’s multiple range tests were performed for each year separately.

**Figure 3:**
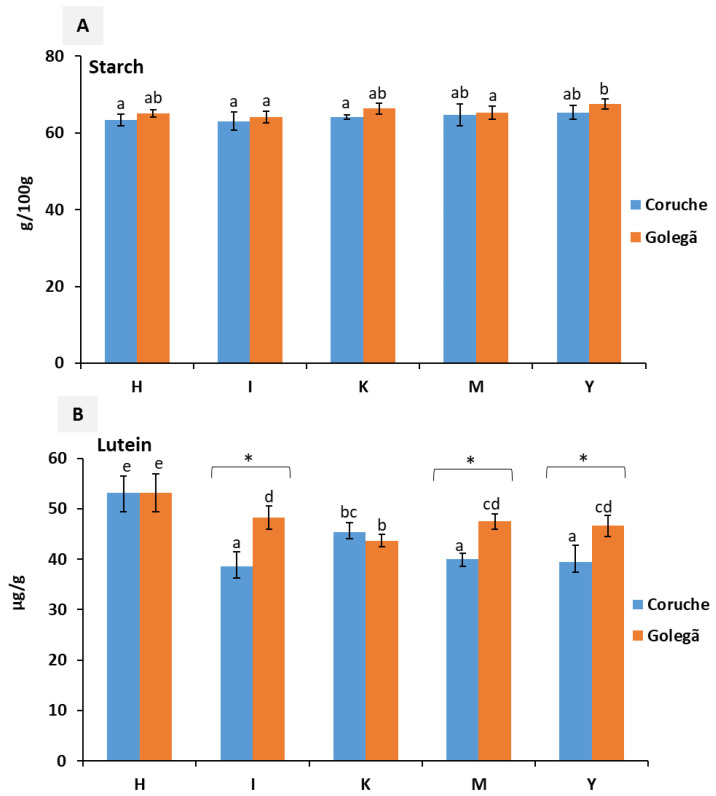
Nutritional composition: (**A**) the starch content and (**B**) the lutein content of five maize varieties collected from the Golegã and Coruche regions. The absence of common letters reveals significant differences at *p* < 0.05, and Tukey’s multiple range tests were performed for each year separately. * indicates a significant difference at *p* < 0.05 between the two harvesting regions.

The nutritional composition of five maize varieties harvested in the Coruche and Golegã regions are described in Figure 1, Figure 2 and Figure 3. Significant differences (*p* < 0.05) were observed in the nutritional composition among the five maize varieties analyzed; this difference may have been influenced by genetic factors, the environmental and climatic conditions, or the type of maize [20].

The protein content ranged from 7.05 to 8.66 g/100 g and from 7.52 to 10.40 g/100 g in the maize varieties from the Coruche and Golegã harvesting regions, respectively (Figure 1); in the literature, the protein content ranged from 8 to 11 g/100 g [21]. Significant differences (*p* < 0.05) were observed between the harvesting regions for the three field samples of the I and M maize varieties (Supplementary Materials Figure S1). In the K and Y varieties, significant differences were observed between the harvesting regions for the I and II field samples (Supplementary Materials Figure S1). The M variety from the Coruche region exhibited the greatest amount of protein (9.68 g/100 g); however, in the Coruche region, the M variety exhibited one of the lowest amounts of protein (7.79 g/100 g). Significant differences between the regions (Coruche and Golegã) were observed for the I, K, and M varieties. The significant differences among the samples and regions of production may have occurred due to the adoption of certain agronomic practices, such as maize–cowpea intercropping [22] and the use of nitrogen fertilization levels > 150 kg N/ha [23], which may have increased the protein content and grain maize quality. Wang [18] reported that the average temperature had the highest indirect effect on the crude protein and fat content. In our study, the greatest protein content was found in the maize harvested from Golegã, at 8.8 g/100 g, with a lower average monthly temperature of 16.9 °C. In Coruche, the average monthly temperature was 18.1 °C and the mean protein content was 7.9 g/100 g. However, the lowest values for the fat content (4.1 g/100 g) were found in maize from the Golegã region. Variations in the responses of the grain starch and crude protein contents to the temperature may have arisen from the differential deposition of starch and protein in endosperm tissues, along with the varying sensitivity of the enzyme systems involved in material transformations to heat stress [24]. In general, the maize varieties growing in the Coruche region showed higher amounts of fat, at 3.86–5.41 g/100 g, compared to the varieties from Golegã (3.11–5.32 g/100 g). This could be explained by the lower levels of precipitation in the Coruche region (53.3 mm) [23,25]. However, the mean fat content among the varieties was similar; the H variety revealed the highest amount (4.87 g/100 g), while the K, M, and Y varieties revealed the lowest values (4.12 g/100 g). In [25], variations between 4 and 6 g/100 g of fat were found in maize grains. The M variety revealed significant differences (*p* < 0.05) between the maize growing regions; these differences in the nutritional quality of maize may have been affected by the use of certain agronomic practices, such as fertilization, crop rotation, or soil conditions [26], as well as the climatic conditions (temperature and precipitation) [18,27], mainly including water stress and higher temperatures, as verified in the Coruche region.

No significant differences were observed in the ash and fiber content between the harvesting regions for the different maize varieties (Figure 2A and Supplementary Materials Figure S2). The ash content ranged from 0.61 to 0.93 g/100 g and from 0.67 to 1.05 g/100 g in the maize varieties from the Coruche and Golegã regions, respectively; emphasized variations of 0.1–4.8 g/100 g were observed in [28]. The M variety showed the highest amount of ash in both harvesting regions, while the H and I varieties revealed lower amounts of ash. Almost 73% of the analyzed samples demonstrated higher amounts of ash in the Golegã region than in Coruche, while in Golegã, around 58% of the samples had a higher fiber content. The maize varieties harvested in Coruche exhibited 1.18–2.40 g/100 g of fiber, and those harvested in the Golegã region exhibited 1.35–2.29 g/100 g (Figure 2B). Slightly higher amounts were detected by the authors of [29], ranging from 3.65 to 5.81 g/100 g. Higher amounts of fiber were found in the H and I varieties, while K and Y exhibited lower fiber amounts.

The amounts of starch ranged from 61.98 to 67.07 g/100 g and from 62.95 to 68.79 g/100 g in the maize samples harvested in Coruche and Golegã, respectively (Figure 3A and Supplementary Materials Figure S3). No significant differences between the harvesting regions were observed in the starch content; however, higher amounts of starch were found in the samples from Golegã, mainly in the K and Y varieties. The highest starch content observed in the maize samples from the Golegã region may be explained by the environmental conditions, mainly the lowest temperature during the active grain-filling period [30]. In Golegã, the temperature ranged from 16.1 to 28.0 °C, while in Coruche, it was 17.2–30.0 °C during the grain-filling period. This led to the formation of smaller starch granules and lower amounts of amylose [25]. Significant differences were observed in around 60% of the tested samples (*p* < 0.05) between the harvesting regions in the lutein content (Figure 3B), mainly for the I, M, and Y varieties. The lutein content in the maize samples from Coruche ranged from 35.48 to 57.23 µg/g, and the content was 43.03–55.59 µg/g in Golegã. These values were slightly higher than those of fortified maize grains, at 0.0–32.3 µg/g according to quantification by high-performance liquid chromatography (HPLC) by the authors of [31], and from 0.87 to 3.13 µg/g of lutein as determined by a spectrophotometer method [29]. The I, M, and Y varieties grown in the Golegã region exhibited amounts of lutein that were around 20% higher than the varieties grown in Coruche. 

### 2.2. Pasting Properties

The pasting properties of the maize varieties and their field triplicates harvested in the Coruche and Golegã regions are described in Table 1.

Significant differences (*p* < 0.05) were observed between the harvesting regions for all the pasting parameters in the maize varieties. The maize flour samples from Coruche revealed a higher peak viscosity (PV), holding strength, and breakdown (BD), whereas the maize samples from Golegã exhibited a higher final viscosity (FV), peak temperature (PT) and setback (SB). In Coruche, the maize varieties showed a lower FV (6598–8704 cP) and PT (71.9–74.6 °C), but a higher PV (5723–8599 cP) and BD (3076–5495 cP) than the varieties from the Golegã harvesting region (Table 2 and Supplementary Materials Table S2). The different behavior of the pasting properties for the varieties between the harvesting regions may have been due to the different environmental conditions in the two regions. However, among the varieties, the Y variety exhibited the highest values for the PV (7121 cP) and FV (9280 cP) and the lowest PT (73.3 °C), while the M variety showed the lowest PV (6047cP) and BD (3187 cP) values and the highest SB (2666 cP). In [28], lower amounts of PV were found, ranging from 2416 to 4570 cP; the BD ranged from 740 to 2059 cP; the FV ranged from 4298 to 5298 cP; and similar levels were found for PT, at 75.1–79.0.

The M variety exhibited FV > PV, indicating that retrogradation occurred more readily due to a higher viscosity during heating, in contrast to varieties with PV > FV, which can readily swell during hydration, but exhibit slower retrogradation post-gelatinization [20]. Nevertheless, a lower FV in maize flour has been associated with a higher protein content and a lower starch content [32], as evidenced by the III sample of the H variety (Supplementary Materials Table S1, Table 1, and Figure 1 and Figure 3). 

The pasting properties of maize flour are related to its potential applications in the food industry. The H and K maize samples, with a lower FV and a higher PV, had excellent potential to be used in foods to form a viscous paste or gel and maintain their texture. A lower PT decreases the spending of energy through food processing. 

### 2.3. Mycotoxin Occurrence

The levels of mycotoxin contamination in the maize varieties harvested in Coruche and Golegã are described in Table 2.

Among all the mycotoxins analyzed, only fumonisins and deoxynivalenol were detected. Significant differences (*p* < 0.05) were observed between the harvest regions in the presence of fumonisin B1 (Fum B1), and no differences were observed for fumonisin B2 (Fum B2) or deoxynivalenol (DON). No differences were detected among the maize samples in the DON occurrence, while for Fum B1, differences were identified among the three samples of each maize variety. The most common mycotoxins in maize are fumonisins, which are found in temperate and Mediterranean regions [15]. Fum B1 is mainly the most toxic and abundant, followed by Fum B2 and B3, in comparison to other grains [33]. This was verified in the present work. Fum B1 was detected in 58% of the maize samples from Coruche, ranging from 176.6 to 2580 µg/kg, and in 33% (173.4–1021.5 µg/kg) of the samples from Golegã. Fum B2 was observed in 33% (99.9–687.9 µg/kg) of the samples harvested in Coruche and in 20% (117.3–201.5 µg/kg) of those harvested from Golegã. Conversely, DON was found in 87% (104.4–144.8 µg/kg) and 80% (102.4–171.0 µg/kg) of the maize samples from Coruche and Golegã, respectively. Similar results have been reported previously; 82% of the maize samples collected from the north of Portugal were found to contain DON, with a mean value of 120 µg/kg, while Fum B1 was found in 41% of the samples, with an average of 400 µg/kg, and Fum B2 was detected in 18% of the maize samples, with a mean value of 100 µg/kg [34]. However, higher levels of mycotoxins were reported between 2012 and 2015 in Serbia, ranging from 88 to 27,103 µg/kg and from 20 to 4651 µg/kg for Fum B1 and Fum B2, respectively. And a variation of DON contamination was observed in 35–100% of all the analyzed samples, with a content of 10–16,350 µg/kg [35]. In 2014–2015, 59.9–9873 µg/kg of Fum B1, 105–9218 µg/kg of Fum B2, and 110–799 µg/kg of DON were found in samples from Albania [36]. In Michigan, Fum B1 was found in 80–96% of the tested samples and ranged from 299.2 to 2179.6 µg/kg; Fum B2 was present in 80–86% of the samples, with a range of 730.7–984.5 µg/kg; and DON ranged from 1228.6 to 5143.1 µg/kg in 93–100% of the samples tested in 2017–2018 [37]. On the other hand, in South Korea, the occurrence of mycotoxins was lower, with Fum B1 (14.2–98.1 µg/kg) and Fum B2 (11.7–75.2 µg/kg) being found in 86% of the tested maize samples [38]. 

In Coruche, the highest Fum B1 and Fum B2 levels occurred in a sample of the H variety, at 2580 ± 53.9 µg/kg and 687 ± 19.2 µg/kg, respectively. Meanwhile, in Golegã, the highest levels were found in a K sample, with 1021 ± 114.2 µg/kg of Fum B1 and 186.2 ± 24.7 µg/kg of Fum B2. The greatest accumulation of DON appeared in a Y sample (144.8 ± 11.1 µg/kg) and an H sample (171.0 ± 0.4 µg/kg) from the Coruche and Golegã growth regions, respectively. Among the maize varieties, the H and Y varieties exhibited the highest mycotoxin contamination, mainly including Fum B1, Fum B2, and DON; however, their concentrations were lower than the maximum permitted levels for the sum of Fum B1 and Fum B2 (4000 µg/kg) and for DON (1750 µg/kg) in unprocessed maize [39,40].

The occurrence of fumonisins is accurately influenced by climatic conditions, particularly the precipitation and temperature during the flowering stage [41], and their production occurs between 15 and 25 °C [42], more specifically under temperatures higher than 23 °C in July and higher than 15.7 °C in October, and under water stress [33]. The higher levels of fumonisins in the maize from the Coruche region may have been due to the temperatures of 26.1 °C in July and 17.9 °C in October and lower amounts of precipitation, in contrast with that observed in Golegã, at 24.1 °C in July and 16.6 °C in October. DON was detected in maize growth in Portugal for the first time in samples harvested in 2020 [43]; before this year, DON had not been found in maize from Portugal [44]. 

### 2.4. Pearson’s Correlation Analysis between Quality Parameters and Climatic Conditions

**Figure 4:**
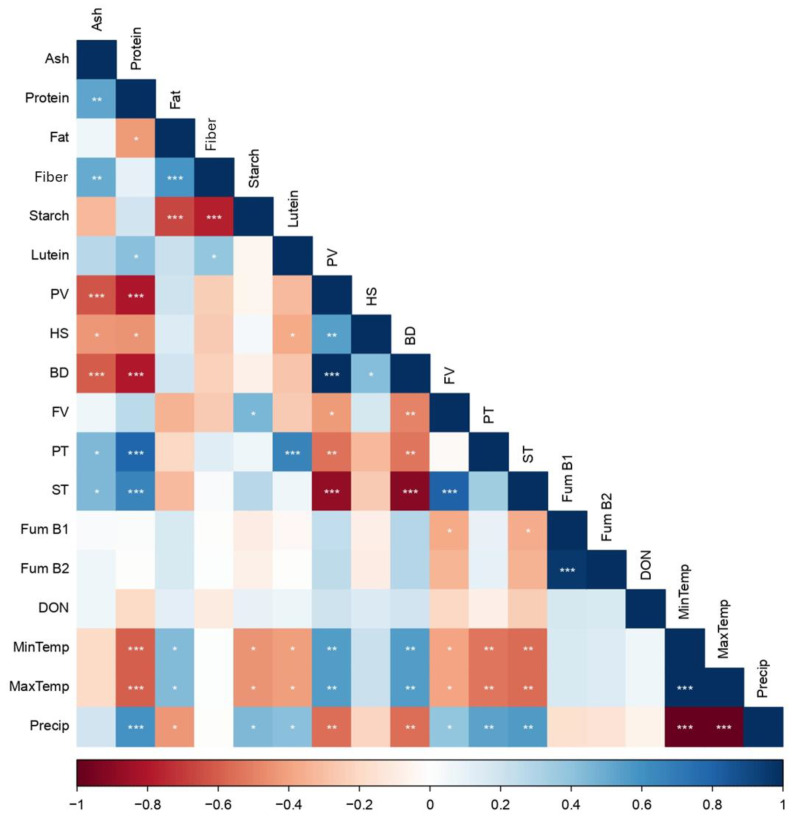
Heat map of correlations between nutritional parameters (ash, protein, fat, fiber, starch, and lutein), pasting properties (PV—peak viscosity, HS—holding strength, BD—breakdown, FV—final viscosity, PT—peak temperature, and ST—setback), and mycotoxin levels (Fum B1—fumonisin B1, Fum B2—fumonisin B2, and DON—deoxynivalenol) of maize as well as climatic condition components (min temp—minimum temperature, max temp—maximum temperature, and precip—precipitation). Statistically significant correlations: * *p* < 0.05, ** *p* < 0.01, *** *p* < 0.001.

The correlation matrix among the nutritional parameters, pasting properties, mycotoxin levels, and climatic conditions is described in Figure 4. The protein content was negatively correlated with the PV and BD, as reported in [45,46], and the temperature (min and max), which was statistically significant (*p* < 0.001). On the other hand, the protein content, the PT, the ST, and the precipitation were positively correlated. The starch and lutein content in maize was negatively influenced by the min and max temperatures, and positively influenced by precipitation (*p* < 0.05). The protein and starch contents decrease under heat stress in the early stages of waxy maize growth [11], and in the grain-filling process [47], a higher temperature holds up the typical process of amyloplast replication and cell division in grains, leading to a reduction in the sink size [11] and the quality of the grains. Almost all of the pasting properties (FV, PT, and ST) were negatively correlated with the min and max temperatures and positively correlated with precipitation (*p* < 0.01); the opposite tendency was observed between the PV and BD with climatic conditions (*p* < 0.01), as observed by Gu et al. [48]. The pasting properties were negatively affected by higher temperatures, even though several authors have reported that these effects are variety-dependent [49]. In the present work, the K and Y varieties were well adapted to high temperatures (>32 °C), such as those observed in the Coruche region, and these varieties exhibited the highest PV, HS, and BD values.

**Figure 5:**
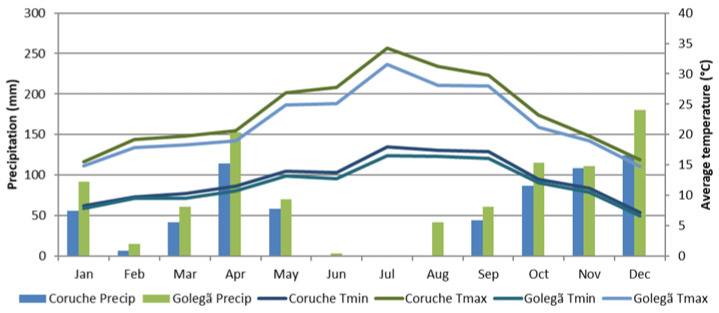
Accumulated monthly precipitation (mm), and average maximumal and minimumal temperatures (°C) registered in 2020 for thein Coruche and Golegã regions.

Negative correlations were verified between fat and starch and between fiber and starch (*p* < 0.001), and positive correlations were observed between ash and protein, between ash and fiber (*p* < 0.01), and between fat and fiber (*p* < 0.001). Statistically significant correlations were monitored between Fum B1 and the pasting properties, mainly the FV and ST. No significant correlations have been verified between mycotoxins and climatic conditions. As anticipated, there is a trend towards the occurrence of toxicogenic molds induced by *Fusarium* mycotoxin production (fumonisins and DON) with higher temperature levels and extreme droughts [50,51,52]. This pattern is evident in maize harvested in the Coruche region, where the temperatures averaged 26.1 °C in July and 17.9 °C in October, accompanied by lower amounts of precipitation (Figure 5). This led to higher average levels of fumonisins (Fum B1, 463.60 µg/kg; Fum B2, 218.25 µg/kg; and DON, 106.66 µg/kg) in comparison to the maize produced in the Golegã region (Fum B1, 218.25 µg/kg; Fum B2, 84.71 µg/kg; and DON, 99.74 µg/kg). In Golegã, fumonisins B1 and B2 were not detected in the H and M varieties, even though the H variety harvested in Coruche (higher temperatures and lower precipitation levels) exhibited the highest levels of fumonisins (1005.4 µg/kg). Nevertheless, the K variety proved to be well adapted to heat stress and lower precipitation levels, there was no occurrence of fumonisins in the Coruche region, and this variety had an excellent nutritional composition and pasting properties. In general, the climatic conditions significantly influenced the nutritional composition and pasting properties of the maize samples.

## 3. Conclusions

Our study evaluated the impact of climatic changes on the nutritional quality, pasting properties, and mycotoxin occurrence in five maize varieties harvested in two regions, Coruche and Golegã, Portugal. The maize samples from Golegã exhibited higher contents of ash, protein, starch, and lutein, while those from Coruche displayed superior pasting parameters such as the PV, HS, and BD. Across all the maize varieties analyzed, the M and H varieties demonstrated a higher nutritional value, particularly in protein, ash, fat, fiber, and lutein, whereas the K variety exhibited notable pasting properties (PV, HS, BD, and PT).

Regarding the climatic conditions, lower average temperatures and higher precipitation levels were associated with increased protein, starch, and lutein contents, as well as an enhanced starch retrogradation ability. Conversely, a positive correlation was observed between the average temperature and the fat content, while a negative correlation was found between the monthly precipitation levels and the PV and BD.

Of the mycotoxins analyzed, only fumonisins B1 and B2 and DON were detected in the maize varieties. DON contamination was found in approximately 87% of the maize samples, while Fum B1 and B2 were detected in 53% and 33% of the maize samples, respectively. Higher levels of mycotoxin contamination were quantified in the maize harvested in the Coruche region, where the average temperatures were higher and the precipitation levels were lower compared to Golegã. However, the levels of mycotoxins did not exceed the regulated limits.

The H and M varieties produced in the Golegã region revealed that fumonisins B1 and B2 were not present. The highest number of fumonisins was detected in the H variety harvested in Coruche, although it showed higher amounts of protein, lutein, and fat. The K variety was the most tolerant of higher temperatures and dry environments, as observed in the Coruche region, exhibiting an excellent quality (higher protein and lutein contents, and a lower fat content) and pasting characteristics (a higher PV and HS, and a lower PT) and no fumonisin contamination.

Our study highlights the crucial role of climatic agronomic conditions in shaping the quality and safety of maize varieties. Furthermore, it emphasizes the significance of incorporating diverse trial sites to gain a comprehensive understanding of how different varieties perform under varying environmental conditions and their effects on maize quality and mycotoxin occurrence.

## 4. Materials and Methods

### 4.1. Sampling

Five yellow maize varieties (H, I, K, M, and Y) were cultivated on two farms in the Coruche and Golegã regions, in the Tagus Valley region of Portugal, in 2020. The agronomic characteristics of the varieties are described in Table 3. This study ran under real farm conditions and a random block design was adopted. Each maize variety was cultivated in triplicate in both regions, and similar agricultural practices were applied in the Coruche and Golegã regions. The regions had a clay soil type, the sowing date was 28 April 2020, the maize was grown in a monoculture, and fertilization was applied as follows: 292 kg N/ha, 92 kg P_2_O_5_/ha, and 120 kg K_2_O/ha. Around 3 kg was harvested from each maize variety, with 17–19% moisture. The maize grains were dried in an oven (Memmert UFB 400, Germany) until reaching a moisture content of 11–13%, and were then carefully mixed and ground in a Retsch rotor mill (SK300) using a 1.00 mm sieve. Each maize flour variety was stored in a plastic cup at −20 °C until the analysis.

### 4.2. Climatic Conditions

The climatic condition data for 2020 are presented in Figure 5. The climatic conditions concerning the monthly accumulated precipitation (mm), maximum temperature (°C), and minimum temperature (°C) were obtained from the E-OBS observational gridded dataset [53]. The daily air temperatures and precipitation amounts were extracted from the gridbox corresponding to Coruche and Golegã.

### 4.3. Nutritional Quality

The maize flour samples were analyzed by following the procedures described by the Association of Official Analytical Chemists (AOAC) to determine the dry matter and ash contents (method 942.05), the total protein (method 954.01), and the total fat (method 920.39). The total starch and dietary fiber analyses were carried out using an assay kit based on AOAC methods 996.11 and 985.29, respectively. The results for the ash, protein, fat, fiber, and starch contents are presented as the grams per hundred grams of sample dry weight (g/100 g dw). The total carotenoid content (TCC) was spectrophotometrically measured at 450 nm according to AACC method 14-60.01 (AACC International, St Paul, MN, USA, 2012). The results are expressed in micrograms of lutein equivalent per gram of sample, as the main carotenoid found in maize.

### 4.4. Pasting Properties

The pasting properties of the maize flour were evaluated by a rapid visco analyzer (RVA) (Newport Scientific, Warriewood, Australia) according to the viscosity profile described in [54]. The maize flour samples, with a solid content of 15%, underwent the following procedure: holding at 50 °C for 2 min, heating to 95 °C for 4.5 min, holding at 95 °C for 4.5 min, cooling to 50 °C for 4 min, and holding at 50 °C for 10 min, with a paddle speed of 960 rpm for the first 10 s and then 160 rpm until the end of the run at 25 min. The following pasting attributes were recorded: the peak viscosity (PV), the holding strength (or minimum) (HS), the breakdown (BD) (calculated as PV-HS), the final viscosity (FV), the peak temperature (PT), and the setback (ST) (calculated as FV-PV). The pasting properties (PV, HS, BD, FV, and ST) were expressed in cPoise (cP) units and the PT was expressed in °C units.

### 4.5. Fumonisin, Zearalenone, Aflatoxin, and Toxin T2 Analyses

The maize flour samples were extracted using acetonitrile 80% (*v*/*v*) in an orbital shaker (Kotterman 4010, Uetze/Hanigsen, Germany) for 1 h. Fumonisins (B1 and B2), zearalenone, aflatoxins (B1, B2, M1, and M2), and toxin T2 were quantified as defined in the method validated by Silva [55]. The analysis was completed using Nexera X2 Shimadzu ultra-high-performance liquid chromatography (UHPLC) coupled with a 5600 time-of-flight mass spectrometry (ToF-MS) detector (SCIEX, Foster City, CA, USA) trained through a Turbo Ion Spray electrospray ionization source operating in the positive mode (ESI). The mobile phase comprised formic acid [A] and acetonitrile [B] at a flow rate of 0.5 mL/min and a sample volume of 20 μL in a Zorbax Eclipse Plus C18 column. The gradient program was as follows: from 90% to 30% [A] in 0–12 min; 30% to 10% [A] in 12–13 min, holding until 14 min; and back to 90% [A] in 14–15 min until the end of the run at 17 min. The acquisition was facilitated by mass spectrometry, and the Analyst^®^TF (SCIEX, Foster City, CA, USA) software was used in a full scan from 100 to 750 Da. The data processing was performed using the PeakView™ and MultiQuant™ (SCIEX, Foster City, CA, USA) software. The limit of detection (LOD) and the limit of quantification (LOQ) of the fumonisins were 62.5 μg/kg and 125 μg/kg, respectively; for the aflatoxins, they were 0.5 μg/kg and 1 μg/kg, respectively; for toxin T2, they were 10 μg/kg and 25 μg/kg, respectively; and for zearalenone, they were 25 μg/kg and 50 μg/kg, respectively.

### 4.6. Deoxynivalenol (DON) Analyses

The previously mentioned LC-ToF-MS method was utilized in the positive mode, making it unsuitable for analyzing deoxynivalenol (DON), which is detected in the negative mode. Using a chemiluminescent biochip immunoassay based on the Evidence Investigator Biochip Array technology (InvestigadorTM EV 4065), as earlier confirmed by Freitas [56], the detection and semi-quantitative screening of deoxynivalenol (DON) in maize samples was carried out. A total of 5 g of maize flour was extracted using 25 mL of acetonitrile/methanol/water (50:40:10, *v*/*v*/*v*), and the extract was then vortexed for 60 s and centrifuged for 10 min at 3000 rpm. Subsequently, the sample was diluted (dilution factor: 75) using a working strength wash buffer (contained in the kit). The diluted sample was transferred to the biochip of the assay Myco 7, which had areas with immobilized antibodies limited to mycotoxins. The screening target concentration was 375 μg/kg of DON.

### 4.7. Statistical Analyses

The nutritional composition, pasting properties, and mycotoxin levels of the maize samples were assessed in triplicate. The data are expressed as the mean ± standard deviation (SD). A one-way analysis of variance (ANOVA), followed by Tukey’s test, was used to assess the significant differences between the samples and the crop field. Differences were considered significant at *p* < 0.05. Statistical analyses were performed using IBM SPSS Statistics 29.0 software (IBM Corporation, New York, NY, USA). To understand the nature and degree of the inter-relationship among the nutritional quality, pasting properties, and mycotoxin occurrence and the climatic condition data, heat mapping of Pearson’s correlations with the corresponding statistical significances was carried out in the software OriginPro 2022 v.9.9.0.225.

## Figures and Tables

**Table 1:** RVA (rapid visco analyzer) parameters of five maize varieties harvested from the Coruche and Golegã regions.

	Location	Peak Viscosity (PV)	Holding Strength (HS)	Breakdown Viscosity (BD)	Final Viscosity (FV)	Pasting Temperature (PT)	Setback Viscosity (SB)
H	Coruche	7258 ± 1205.1 ^cd^	2750 ± 88.4 ^ab^	4508 ± 1124.9 ^cd^	6702 ± 152.5 ^a^*	74.5 ± 0.3 ^cd^	556 ± 1272.7 ^a^
	Golegã	6547 ± 1281.3 ^bc^	2845.2 ± 193.1 ^b^	3702 ± 1090.6 ^bc^	7941 ± 560.2 ^b^	74.1 ± 0.7 ^c^	1394 ± 808.7 ^abc^
I	Coruche	7208 ± 115.6 ^cd^	3011 ± 75.0 ^c^	4197 ± 104.8 ^cd^	81,623 ± 106.0 ^bc^	72.9 ± 0.4 ^ab^*	955 ± 89.6 ^abc^
	Golegã	6680 ± 886.3 ^bc^	3070 ± 74.3 ^c^	3610 ± 905.3 ^bc^	7861 ± 517.9 ^ab^	74.0 ± 0.5 ^c^	1181.8 ± 1324.5 ^abc^
K	Coruche	7899 ± 671.0 ^d^*	3181 ± 83.1 ^d^*	4718 ± 62.4 ^d^*	7787 ± 313.1 ^ab^	73.9 ± 0.0 ^c^	112 ± 897.1 ^a^*
	Golegã	5963 ± 851.3 ^b^	2850 ± 104.2 ^b^	3114 ± 778.3 ^ab^	8777 ± 342.5 ^bc^	74.4 ± 0.5 ^cd^	2814 ± 836.0 ^cd^
M	Coruche	7108 ± 295.2 ^cd^*	2990 ± 66.1 ^c^*	4119 ± 293.6 ^cd^*	8657 ± 140.5 ^bc^	73.2 ± 0.5 ^b^*	1549 ± 361.6 ^abcd^
	Golegã	4985 ± 362.2 ^a^	2729 ± 136.3 ^a^	2256 ± 450.1 ^a^	8768 ± 657.8 ^bc^	74.9 ± 0.7 ^d^	3783 ± 928.1 ^d^
Y	Coruche	7466 ± 906.1 ^cd^	2990 ± 91.7 ^c^	4477 ± 979.3 ^cd^	7861 ± 803.8 ^ab^*	72.4 ± 1.0 ^a^*	394.2 ± 1666.0 ^ab^
	Golegã	6775 ± 862.8 ^bc^	3066 ± 32.7 ^c^	3708 ± 867.7 ^bc^	9280 ± 1364.6 ^c^	74.1 ± 0.7 ^c^	2505 ± 2171.8 ^bcd^

* indicates a significant difference at *p* < 0.05 between the harvest regions. The absence of common letters reveals significant differences at *p* < 0.05, and Tukey’s multiple range tests were performed for each year separately.

**Table 2:** Occurrence of mycotoxins in the five maize varieties collected from the Coruche and Golegã regions.

	Location	Fumonisin B1 (Fum B1)	Fumonisin B2 (Fum B2)	Deoxynivalenol (DON)
H	Coruche	1005.4 ± 1244.4 ^b^*	271.0 ± 334.1 ^b^	114.3 ± 14.6 ^ab^
	Golegã	<LOD	<LOD	144.1 ± 25.6 ^c^
I	Coruche	196.3 ± 207.3 ^a^	75.0 ± 30.5 ^a^	118.1 ± 22.1 ^abc^
	Golegã	390.9 ± 384.1 ^ab^	108.8 ± 77.1 ^ab^	123.5 ± 29.3 ^abc^
K	Coruche	<LOD	<LOD	106.7 ± 7.7 ^a^
	Golegã	419.1 ± 472.1 ^ab^	103.7 ± 64.8 ^ab^	112.3 ± 28.0 ^ab^
M	Coruche	549.4 ± 556.7 ^ab^	214.7 ± 133.6 ^ab^	119.6 ± 15.8 ^abc^
	Golegã	<LOD	<LOD	105.6 ± 6.6 ^a^
Y	Coruche	438.9 ± 289.4 ^ab^	95.5 ± 36.3 ^a^	136.0 ± 18.9 ^bc^
	Golegã	238.5 ± 286.1 ^a^	80.8 ± 44.7 ^a^	116.2 ± 19.0 ^abc^

* indicates a significant difference at *p* < 0.05 between the harvest regions. The absence of common letters reveals significant differences at *p* < 0.05, and Tukey’s multiple range tests were performed for each year separately.

**Table 3:** Agronomic characteristics of maize varieties.

Maize	Type of Hybrid	Grain Color	Type of Grain	Ability	Cycle Type	FAO Classification
H	Single-cross	Yellow	Dent	Double	Long	600
I	Single-cross	Yellow	Dent	Double	Long	600
K	Single-cross	Yellow	Dent	Double	Medium	600
M	Single-cross	Yellow	Dent	Grain	Medium	500
Y	Single-cross	Yellow	Dent	Double	Medium	500

## Data Availability

The data presented in this study are available in this article or Supplementary Material.

## Supplementary Materials

The following supporting information can be downloaded at: https://www.mdpi.com/article/10.3390/toxins16060275/s1, Figure S1: Nutritional composition: A—the protein content and B—the fat content of three replications of five maize varieties collected from the Golegã and Coruche regions. The absence of common letters reveals significant differences at *p* < 0.05, and Tukey’s multiple range tests were performed for each year separately. * indicates a significant difference at *p* < 0.05 between the two harvesting regions; Figure S2: Nutritional composition: A—the ash content and B—the fiber content of three replications of five maize varieties collected from the Golegã and Coruche regions. The absence of common letters reveals significant differences at *p* < 0.05, and Tukey’s multiple range tests were performed for each year separately. * indicates a significant difference at *p* < 0.05 between the two harvesting regions; Figure S3: Nutritional composition: A—the starch content and B—the lutein content of three replications of five maize varieties collected from the Golegã and Coruche regions. The absence of common letters reveals significant differences at *p* < 0.05, and Tukey’s multiple range tests were performed for each year separately. * indicates significant differences at *p* < 0.05 between the two harvesting regions; Table S1: RVA (rapid visco analyzer) parameters, including three replications of five maize varieties harvested from the Coruche and Golegã regions; Table S2: Occurrence of mycotoxins in the three replications of five maize varieties collected from the Coruche and Golegã regions.
