# Supplementary material for: Comparative Analysis of Maize Physico-Chemical Parameters and Mycotoxin Levels in Dual Environments
Source: Toxins (Basel). 2024 Jun 17;16(6):275. doi: 10.3390/toxins16060275 (PMC11209266; doi:10.3390/toxins16060275)
Supplement: Supplementary file 1 [file toxins-16-00275-s001.zip › toxins-3016596-supplementary.pdf]

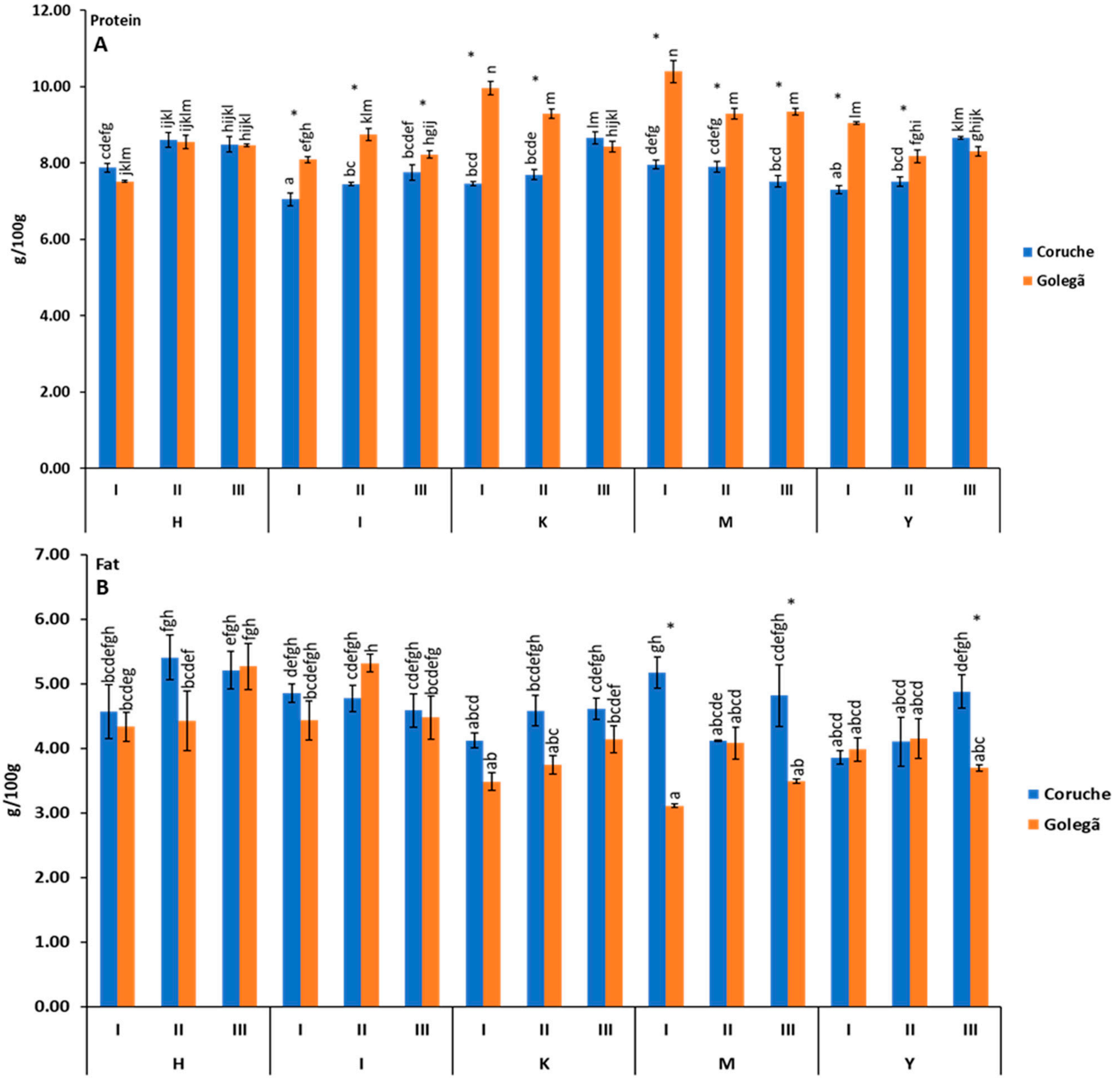

Figure S1. Nutritional composition: **(A)** protein content and **(B)** fat content in the three replications of five maize varieties collected from Golegã and Coruche region. The absence of common letters reveals significant differences at $p < 0.05$, and Tukey's multiple range tests were performed for each year separately. * indicates a significant difference at $p < 0.05$ between the two harvesting regions.

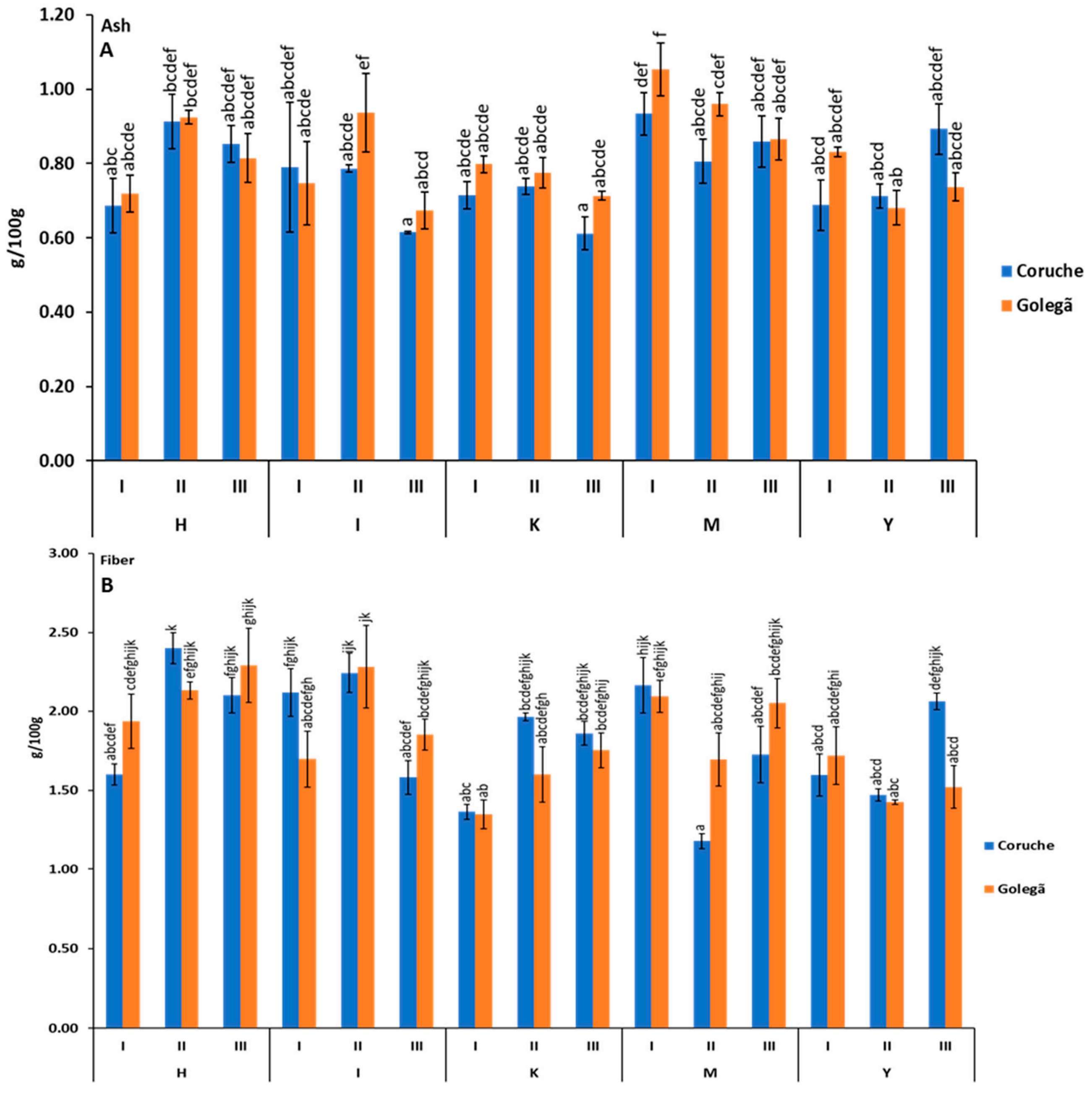

Figure S2. Nutritional composition: **(A)** ash content and **(B)** fiber content in the three replications of five maize varieties collected from Golegã and Coruche region. The absence of common letters reveals significant differences at $p < 0.05$, and Tukey's multiple range tests were performed for each year separately.

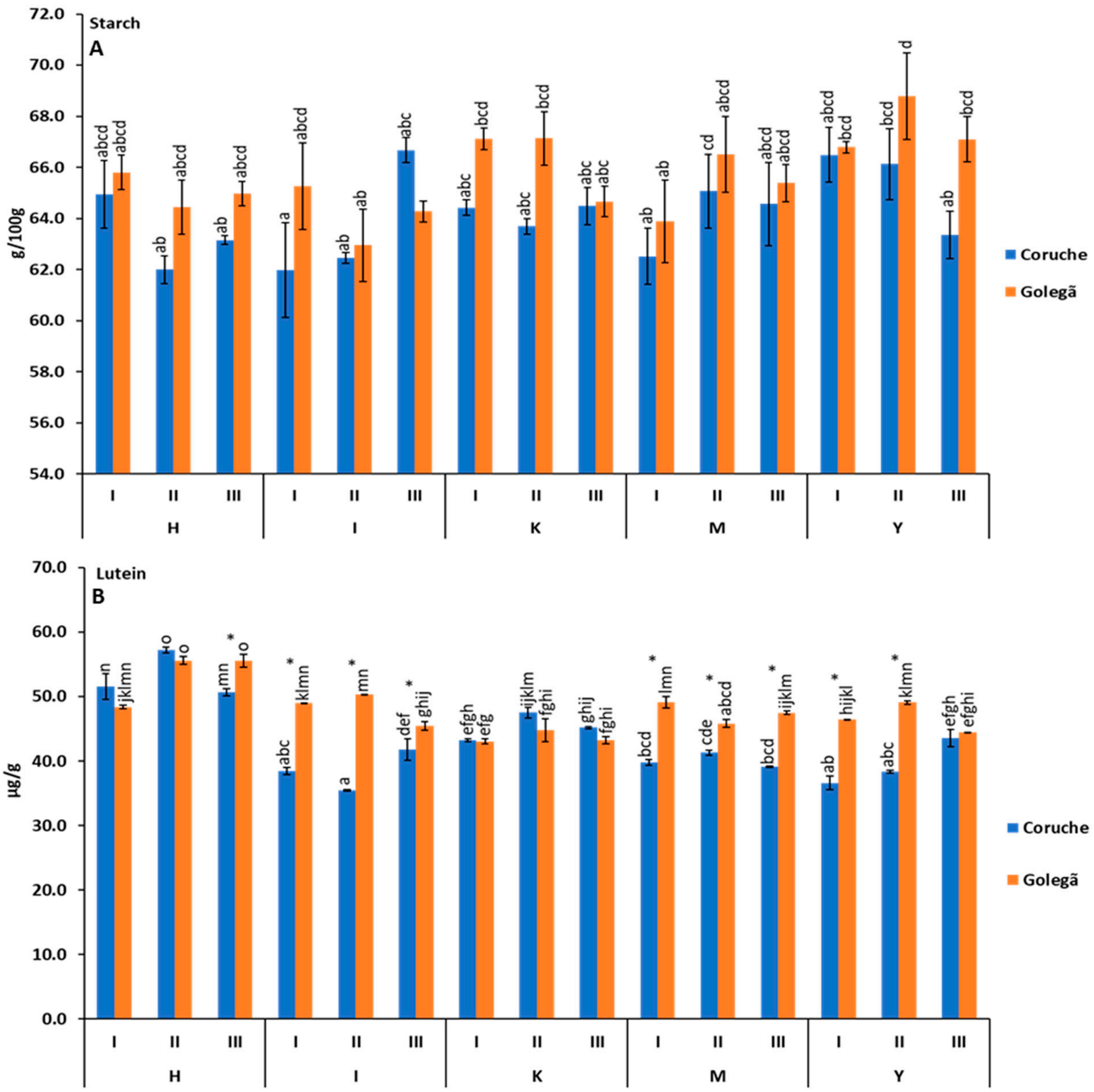

Figure S3. Nutritional composition: **(A)** starch content and **(B)** lutein content in the three replications of five maize varieties collected from Golegã and Coruche region. The absence of common letters reveals significant differences at $p < 0.05$, and Tukey's multiple range tests were performed for each year separately. * indicates a significant difference at $p < 0.05$ between the two harvesting regions.

Table S1. RVA (rapid visco analyzer) parameters in the three replications of five maize varieties harvested from the Coruche and Golegã regions

		Peak Viscosity (PV)		Trough (Holding Strength) (HS)		Breakdown Viscosity (BD)		Final Viscosity (FV)		Pasting Temperature (PT)		Setback Viscosity (SB)	
		Coruche	Golegã	Couche	Golegã	Coruche	Golegã	Coruche	Golegã	Coruche	Golegã	Coruche	Golegã
H	I	8220±151.3^{opq}	8178±31.8^{opq}	2802±72.8^{bcdefg*}	3090±39.6^{ijklm}	5418±78.5^{p}	5088±7.8^{op}	6598±268.0^{a*}	8636±36.1^{jk}	74.6±0.0^{def}	73.2±0.0^{abcd}	-1622±116.7^{a*}	459±4.2^{f}
	II	5723.5±48.8^{cde}	5494±82.7^{bcde}	2647±39.6^{ab}	2702±57.3^{abc}	3076±88.4^{def}	2792±25.4^{bcde}	6780±72.8^{a*}	7757±101.8^{defg}	74.3±0.5^{cdef}	74.5±0.1^{def}	1056±121.6^{ghij*}	2264±19.1^{no}
	III	7832±136.5^{mno*}	5971±7.1^{efg}	2801±15.6^{bcdefg}	2744±5.6^{abcde}	5030±120.9^{op*}	3227±1.4^{ef}	6728±65.8^{a*}	7430±29.0^{cd}	74.5±0.0^{def}	74.6±0.1^{def}	-1103±70.7^{bc*}	1460±21.9^{ijkl}
I	I	7124±65.8^{jk*}	7790±154.8^{lmno}	2916±3.5^{defghij}	3066±31.8^{ijklm}	4208±62.2^{ijkl*}	4724±123.0^{mno}	8044±50.2^{efgh*}	7266±92.6^{bc}	72.6±0.0^{ab*}	74.2±0.5^{cdef}	919±15.6^{fgh*}	-525±62.2^{de}
	II	7287±186.7^{jklmn*}	5908±82.7^{efg}	3040±4.9^{hijkl}	3151±5.6^{lm}	4248±191.6^{ijklm*}	2756±77.1^{bcde}	8274±0.7^{ghij}	7902±55.9^{efgh}	72.9±0.4^{abc}	74.2±0.6^{cdef}	986±187.4^{ghij*}	1995±138.6^{mn}
	III	7212±35.4^{jklm*}	6340±78.5^{fgh}	3076±2.1^{ijklm}	2991±31.1^{fghijk}	4136±37.5^{ijk*}	3350±47.4^{fgh}	8170±23.3^{fghi}	8416±28.3^{hij}	73.3±0.0^{abcd}	73.6±0.5^{bcde}	958±12.0^{fghi*}	2076±50.2^{mn}
K	I	7935±328.1^{nop*}	5060±119.5^{ab}	3139±97.6^{klm*}	2719±24.0^{abcd}	4796±230.5^{no*}	2342±95.4^{b}	7596±303.3^{cdef*}	8456±28.3^{ijk}	73.9±0.0^{bcde}	74.8±0.6^{ef}	-340±24.7^{e*}	3396±91.2^{rs}
	II	8599±104.6^{q*}	5881±165.5^{efg}	3245±106.1^{m*}	2932±36.1^{efghij}	5354±1.4^{p*}	2948±129.4^{cdef}	7622±89.8^{cdef*}	9190±149.2^{l}	74.0±0.0^{bcde}	74.6±0.0^{def}	-978±14.8^{cd*}	3308±16.2^{qr}
	III	7163±253.1^{jkl}	6948±26.2^{ijk}	3160±33.9^{lm*}	2897±5.6^{cdefghi}	4003±219.2^{ij}	4050±20.5^{ij}	8144±79.9^{fghi*}	8684±2.1^{jk}	73.9±0.0^{bcde}	74.0±0.0^{bcde}	983±173.2^{ghij*}	1737±24.0^{klm}
M	I	6792±286.4^{hij*}	5210±39.6^{bcd}	2976±33.2^{fghijk*}	2740±2.1^{abcde}	3817±319.6^{hi*}	2470±37.5^{bc}	8688±170.4^{jk*}	7950±31.8^{defg}	73.3±0.0^{abcd*}	75.6±0.5^{f}	1895±456.8^{lmn*}	2740±71.4^{op}
	II	7226±135.1^{jklm*}	4548±86.3^{a}	3051±24.0^{hijklm}	2860±67.2^{cdefghi}	4176±111.0^{ijkl*}	1688±19.1^{a}	8704±71.4^{jk*}	9354±153.4^{l}	73.6±0.5^{bcde}	74.9±0.5^{ef}	1477±206.5^{jkl*}	4806±67.2^{s}
	III	7306±171.8^{jklmn*}	5197±271.5^{bc}	2942±87.7^{fghijk*}	2588±117.4^{a}	4364±84.1^{jklmn*}	2609±154.1^{bcd}	8581±216.4^{ijk}	9000±92.6^{kl}	72.8±0.5^{abc}	74.2±0.4^{cdef}	1274±44.5^{hijk*}	3804±178.9^{t}
Y	I	8445±106.1^{pq*}	5800±105.4^{efg}	2950±25.4^{ghijk}	3086±47.4^{ijklm}	5495±80.6^{p*}	2713±58.0^{bcd}	6836±47.4^{ab*}	10295±41.0^{m}	71.9±0.0^{a*}	74.6±0.0^{def}	-1608±58.7^{ab*}	4496±146.4^{t}
	II	7524±142.1^{klmno*}	6800±19.1^{hij}	2916±38.9^{defghij}	3038±17.7^{hijkl}	4609±181.0^{klmno*}	3763±1.4^{ghi}	8244±44.5^{ghij*}	10018±28.3^{m}	71.9±0.8^{a}	73.2±0.1^{abcd}	720±97.6^{fg*}	3218±9.2^{pq}
	III	6430±31.8^{ghi*}	7724±57.3^{klmno}	3103±7.1^{jklm}	3075±12.7^{ijklm}	3326±38.9^{fg*}	4649.5±44.5^{lmno}	8500±122.3^{hij*}	7526±46.0^{cde}	73.6±0.5^{bcde}	74.5±0.0^{def}	2071±90.5^{mn*}	-199±11.3^{e}

Table S2. Occurrence of mycotoxins in the three replications of five maize varieties collected from the Coruche and Golegã regions

		Fumonisin B1		Fumonisin B2		Deoxynivalenol	
		Coruche	Golegã	Coruche	Golegã	Coruche	Golegã
	I	259.0 ± 45.8^{abc}	<LOD	<LOD	<LOD	132.0 ± 4.3	171.0 ± 0.4
H	II	176.6 ± 0.4^{ab}	<LOD	<LOD	<LOD	106.4 ± 9.1	124.5 ± 8.9
	III	2580 ± 53.9^{g*}	<LOD	687.9 ± 19.2^{c}	<LOD	104.4 ± 2.7	136.6 ± 29.4
	I	<LOD	857.1 ± 22.2^{def*}	<LOD	201.5 ± 63.0^{ab}	<LOD	109.9 ± 14.0
I	II	<LOD	<LOD	<LOD	<LOD	122.4 ± 15.2	100.8 ± 1.1
	III	463.9 ± 1.3^{abcde*}	253.0 ± 9.0^{abc}	99.9 ± 5.8^{a}	<LOD	131.8 ± 34.0	159.8 ± 7.8
	I	<LOD	1021.5 ± 114.2^{ef*}	<LOD	186.2 ± 24.7^{ab}	108.9 ± 12.6	137.1 ± 45.5
K	II	<LOD	<LOD	<LOD	<LOD	111.1 ± 1.4	<LOD
	III	<LOD	173.4 ± 20.7^{ab*}	<LOD	<LOD	<LOD	<LOD
	I	1248 ± 97.3^{f*}	<LOD	355.3 ± 40.8^{b}	<LOD	112.6 ± 6.8	109.4 ± 3.5
M	II	<LOD	<LOD	<LOD	<LOD	108.5 ± 1.0	107.3 ± 10.4
	III	337.6 ± 4.7^{abc*}	<LOD	226.2 ± 37.0^{ab}	<LOD	137.8 ± 14.1	<LOD
	I	735.0 ± 19.7^{cde*}	<LOD	99.0 ± 5.2^{a}	<LOD	140.1 ± 9.2	105.7 ± 1.5
Y	II	<LOQ	<LOD	<LOD	<LOD	122.8 ± 32.3	102.4 ± 0.9
	III	456.5 ± 86.1^{abcde}	590.4 ± 19.5^{bcde}	<LOQ	117.3 ± 7.7^{a}	144.8 ± 11.1	140.6 ± 4.2
